# Morphological characterization of large intercalated neurons provides novel insight on intrinsic networks of the amygdala

**DOI:** 10.1186/1471-2210-11-S2-A9

**Published:** 2011-09-05

**Authors:** Daniela Busti, Thomas Bienvenu, Ben Micklem, Peter J Magill, Ryuichi Shigemoto, Marco Capogna, Francesco Ferraguti

**Affiliations:** 1 Department of Pharmacology, Innsbruck Medical University, 6020 Innsbruck, Austria; 2 MRC, Anatomical Neuropharmacology Unit, Oxford OX1 3TH, United Kingdom; 3 Division of Cerebral Structure, National Institute for Physiological Sciences, Okazaki 444-8787, Japan

## Background

Although extinction-based therapies are effective treatments for anxiety disorders, the neural bases of fear extinction remain still largely unclear. Recent evidence suggests that the intercalated cell masses of the amygdala (ITCs) are critical structures for fear expression and extinction. They consist of clusters of densely packed medium spiny GABAergic neurons surrounding the basolateral amygdaloid complex (BLA). Five percent of ITC neurons are large cells mostly present near the cluster borders. So far, no information is available regarding the neurochemical features, afferents and efferents of large ITC cells, preventing any elucidation of their functional role. Only recently we discovered that large ITC neurons display immunoreactivity for either neurokinin 1 or metabotropic glutamate 1α (mGlu1α) receptors. We also found that dendrites of these neurons receive inhibitory inputs from medial capsular projecting ITC cells [1]. The aim of our study consists in the characterization of the morphological features, as well as the afferent and efferent connectivity, of large ITC neurons in order to further clarify their potential participation in the neuronal processes underlying fear extinction.

## Methods

The neurochemical phenotype of large ITC neurons and their afferent connectivity were investigated by confocal and pre-embedding electron microscopy performed on both rat amygdala slices and on three large mGlu1α-positive ITC neurons, recorded and filled with neurobiotin in rat by means of the juxtacellular technique *in vivo*. In addition, by Neurolucida, we could reconstruct the full dendritic and axonal arborization of one large filled ITC neuron.

## Results

Immunofluorescence analysis demonstrated that large ITC mGlu1α-positive neurons express the α1 subunit of GABA_A_ receptors and the calcium-binding protein parvalbumin. The dendrites of these large ITC neurons were decorated by axon terminals enriched in presynaptic mGlu7 and/or mGlu8 receptors which, as shown by electron microscopy analysis, established both excitatory and inhibitory synapses. The full tridimensional reconstruction of one *in vivo*-recorded large ITC neuron showed a very wide axonal arborization predominantly innervating the BLA but also extending, rostrally, to the dorsal endopiriform nucleus and, caudally, to the entorhinal cortex.

## Conclusions

These findings elucidate for the first time some of the key anatomical features of the large ITC neurons and shed new light on intrinsic microcircuits of the amygdala containing both pre- and post-synaptic mGlu receptors. Pharmacological manipulation of these receptors may thus influence extinction of fear conditioning and represent a new therapeutic avenue for anxiety disorders.

## References

[B1] Busti D Geracitano R Whittle N Dalezios Y Mańko M Kaufmann WA Sätzler K Singewald N Capogna M Ferraguti F Different fear states engage distinct networks within the intercalated cell clusters of the amygdala J Neurosci 2011 31 5131 5144 10.1523/JNEUROSCI.6100-10.2011 21451049 PMC6622967

